# Epitope mapping of spontaneous autoantibodies to anaplastic lymphoma kinase (ALK) in non-small cell lung cancer

**DOI:** 10.18632/oncotarget.21182

**Published:** 2017-09-23

**Authors:** Mark M. Awad, Cristina Mastini, Rafael B. Blasco, Luca Mologni, Claudia Voena, Lara Mussolin, Stacy L. Mach, Anika E. Adeni, Christine A. Lydon, Lynette M. Sholl, Pasi A. Jänne, Roberto Chiarle

**Affiliations:** ^1^ Lowe Center for Thoracic Oncology, Department of Medical Oncology, Belfer Center for Applied Cancer Science, Dana-Farber Cancer Institute, Harvard Medical School, Boston, MA, USA; ^2^ Department of Molecular Biotechnology and Health Sciences, University of Turin, Turin, Italy; ^3^ Department of Pathology, Children's Hospital, Harvard Medical School, Boston, MA, USA; ^4^ Department of Health Sciences, University of Milano-Bicocca, Milan, Italy; ^5^ Department of Women and Children's Health, University of Padua, Padua, Italy; ^6^ Department of Pathology, Brigham and Women's Hospital, Harvard Medical School, Boston, MA, USA

**Keywords:** lung cancer, anaplastic lymphoma kinase, autoantibodies, immunotherapy

## Abstract

The anaplastic lymphoma kinase (ALK) is recognized by the immune system as a tumor antigen, and preclinical evidence suggests that ALK-rearranged NSCLCs can also be successfully targeted immunologically using vaccine-based approaches. In contrast to ALK-rearranged lymphomas, the frequency and clinical significance of spontaneous ALK immune responses in patients with ALK-rearranged NSCLCs are largely unknown. We developed an enzyme-linked immunosorbent assay (ELISA) to measure anti-ALK antibody levels and mapped specific peptide epitope sequences within the ALK cytoplasmic domain in patients with non-small cell lung cancer. The ELISA method showed good correlation with ALK antibody titers measured with a standard immunocytochemical approach. Strong anti-ALK antibody responses were detected in 9 of 53 (17.0%) ALK-positive NSCLC patients and in 0 of 38 (0%) ALK-negative NSCLC patients (P<0.01), and the mean antibody levels were significantly higher in ALK-positive than in ALK-negative NSCLC patients (P=0.02). Across individual patients, autoantibodies recognized different epitopes in the ALK cytoplasmic domain, most of which clustered outside the tyrosine kinase domain. Whether the presence of high ALK autoantibody levels confers a more favorable prognosis in this patient population warrants further investigation.

## INTRODUCTION

About 3-7% of non-small cell lung cancers (NSCLC) harbor rearrangements in the anaplastic lymphoma kinase (*ALK*) gene [1]. For ALK-positive NSCLC, treatment with the tyrosine kinase inhibitor (TKI) crizotinib results in a high objective response rate (ORR) of ∼60%, but the median progression-free survival (mPFS) is only 8-10 months [2], owing to the rapid emergence of acquired drug resistance through a variety of mechanisms [3–6]. Among crizotinib-resistant patients, the objective response rate to the FDA-approved next-generation ALK inhibitors ceritinib and alectinib is 48-56%, with a mPFS of only 7.0-8.1 months [7–9] because of the invariable development of drug resistance [10, 11]. While ALK TKIs have had a major impact on lung cancer care, novel therapeutic approaches for ALK-positive cancers are necessary to provide safe and durable responses for patients.

A potential alternative strategy for treating ALK-positive cancers is to exploit the natural immune responses against tumor cells expressing ALK protein. Immune system recognition of the ALK protein has been demonstrated in patients with ALK-positive anaplastic large cell lymphoma (ALCL). For example, ALK autoantibodies can be detected from patient serum, and pretreatment ALK antibody titers are inversely correlated with stage of disease, amount of circulating tumor cells, and cumulative incidence of relapse [12]. Furthermore, ALK-specific tumor-reactive T-cells can be detected in the blood of ALK-positive ALCL patients, but not in healthy volunteers [13]. Finally, we have shown that high ALK autoantibody titers in ALK-positive ALCL patients are associated with a favorable prognosis [14].

The potential therapeutic benefit of an anti-ALK immune response has been demonstrated in a mouse model of ALK-positive ALCL, in which a DNA-based ALK vaccine was shown to generate ALK-specific cytotoxic T-cell responses and protect mice from developing lymphoma [15]. This ALK vaccine was also recently shown to be highly effective in a model of ALK-positive NSCLC. Mice prophylactically treated with vaccine were protected from developing lung tumors after being challenged with ALK-positive tumor cells. Furthermore, in transgenic mice expressing EML4-ALK under a lung-specific promoter, treatment with an ALK vaccine after lung tumors had already formed significantly reduced tumor growth and extended survival in vaccinated mice compared to control mice [16].

A recent report on a small series of cases showed that ALK-positive NSCLC patients can develop anti-ALK immune responses [17], but a detailed characterization of these autoantibodies and their potential clinical implications in ALK-positive NSCLC are unknown. The existing approach for detecting endogenous antibodies against ALK in lymphoma patients is based on a semi-quantitative immunocytochemical technique. In this method, serum from a patient is used as source of anti-ALK primary antibodies to coat COS cells transiently transfected to overexpress an NPM-ALK fusion protein. Semi-quantitative measurements are then obtained by serial dilutions of the patient's serum [18]. This approach is subjective because it relies on the evaluation of the positivity of the immunostains by observers. To overcome these limitations, we developed a novel enzyme-linked immunosorbent assay (ELISA) to rapidly and quantitatively detect and measure ALK-specific antibodies in the serum of patients with ALK-positive NSCLC. Furthermore, we mapped the ALK epitopes that induce the immune responses and we show that the presence of ALK immune response might have prognostic impact in ALK-positive NSCLC patients.

## RESULTS

### Detection of autoantibodies in ALK-positive NSCLC

**Figure 1:**
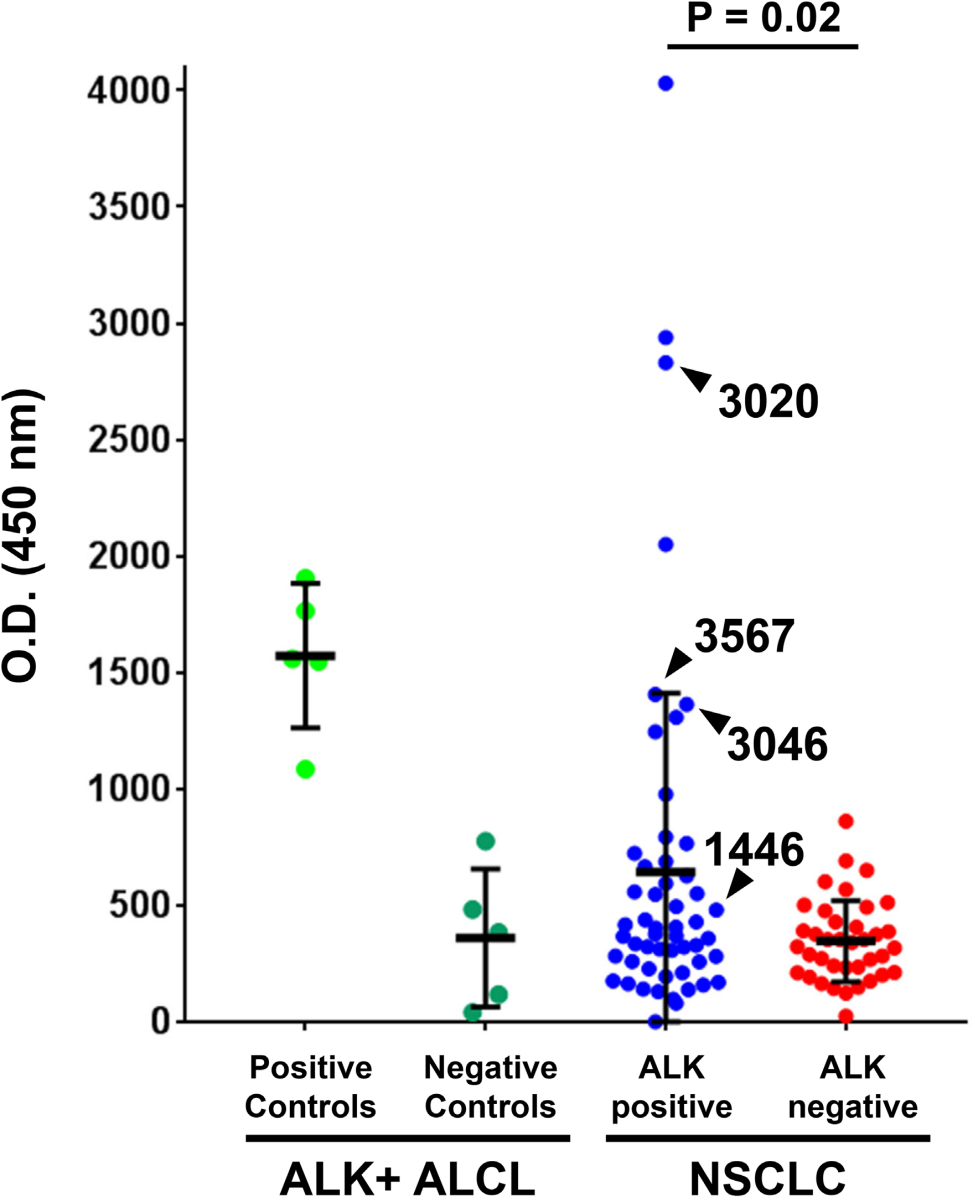
A subset of ALK-positive non-small cell lung cancer (NSCLC) patients have high serum ALK autoantibodies The ALK cytoplasmic domain (amino acids 1064-1620) was used to coat ELISA plates. ELISA O.D. values are shown using serum from ten patients with ALK-positive anaplastic large cell lymphoma (ALCL) from a previously reported series [14], 53 with ALK-positive NSCLC, and 38 with ALK-negative NSCLC. For each group, the median and standard deviation are shown. Samples used for subsequent Western blot analysis (for Figure 2) are delineated with arrowheads.

A new enzyme-linked immunosorbent assay (ELISA) was first developed to detect circulating ALK autoantibodies in the serum of cancer patients. A recombinant ALK protein encompassing amino acids 1064-1620 of the cytoplasmic portion of ALK (Supplementary Figure 1) was synthesized, purified, and then directly coated onto ELISA plates. To validate this assay, we selected five samples from our previously-published series of ALK-positive ALCL patients who were known to have high ALK autoantibody titers, as well as five low/negative ALCL with titers <1:750 as detected using an immunocytochemical approach [12, 14, 18]. Samples from ALK-positive ALCL patients who were known to have high ALK autoantibodies showed distinctly higher optical density (O.D.) values than patients known to have low ALK antibodies (Figure 1). Despite the limited number of samples in this validation set, there was a significant correlation between the titers obtained with the immunocytochemical technique and our ELISA assay (Supplementary Figure 2).

Blood was collected from 53 ALK-positive stage IV NSCLC patients and 38 ALK-negative stage IV NSCLC patients during the course of routine clinical care at our institution. Among the patients with ALK-rearranged NSCLC, 17 were TKI-naïve and 36 had been exposed to at least one ALK TKI at the time of blood collection. ALK-positive NSCLC patients had a significantly higher mean O.D. level (range 0 to 4031, mean ± SD = 643.4 ± 767.8) compared to ALK-negative NSCLC patients (range 24 to 861, mean ± SD = 346.8 ± 174.9, P = 0.02), as shown in Figure 1. High ALK autoantibody levels (defined in this study as an O.D. value greater than three standard deviations above the mean among ALK negative NSCLC cases, i.e. > O.D. 871.5) were detected in 9 of 53 (17.0%) ALK-positive NSCLC cases and 0 of 38 (0%) ALK-negative NSCLC cases (P < 0.01). Among the 26 ALK-positive NSCLC patients in whom at least 3 serial serum samples were available, antibody levels typically did not greatly vary over time within an individual (Supplementary Figure 3).

**Figure 2:**
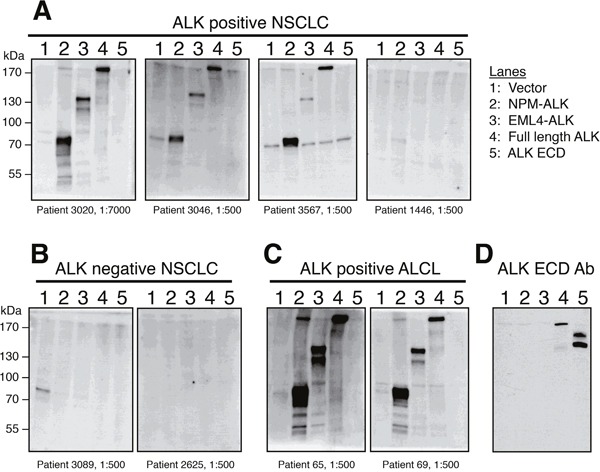
Western blot analysis confirms that ALK-positive NSCLC patient serum specifically recognizes the ALK intracellular domain (ICD) **(A)** Three ALK-positive NSCLC patients with high (patients 3020, 3567, and 3026) ELISA O.D. values were analyzed by Western blot and showed strong reactivity to NPM-ALK, EML4-ALK, and recombinant full-length ALK, but not to the extracellular domain (ECD) of ALK which is not included in ALK fusions. Serum from one ALK-positive NSCLC patient (patient 1446, A) and two ALK-negative NSCLC patients **(B)** with low ELISA O.D. values did not detect any of the ALK proteins by Western blot. **(C)** Western blots using serum from two ALK-positive ALCL patients are shown as a positive control. **(D)** A monoclonal antibody raised against the ALK extracellular domain (ECD) [28] detects only full-length ALK and the ALK ECD, but not the fusion proteins NPM-ALK or EML4-ALK.

We validated our ELISA results by using patient serum as the primary antibody in Western blot analysis. Serum from three ALK-positive NSCLC patients with high ALK autoantibody titers (patients 3020, 3046, 3567) was incubated with lysates from cells expressing NPM-ALK, EML4-ALK, and full-length ALK and detected bands at the expected sizes. Importantly, patient serum did not recognize the extracellular or transmembrane ALK domains (encoded by exons 1-19), which are not part of the expressed oncogenic fusion protein in ALK-positive NSCLC (Figure 2). As expected, serum from ALK-positive ALCL patients with high autoantibody titers by ELISA also specifically recognized only the C-terminal rearranged portion of ALK. Conversely, in one ALK-positive and two ALK-negative lung cancer patients with low anti-ALK levels by ELISA, Western blot showed no detectable bands (Figure 2). We further confirmed that these autoantibodies were specific for the ALK intracellular domain using Western blots with lymphoma cells where the expression of the endogenous NPM-ALK was knocked down with a doxycycline-inducible ALK shRNA (Supplementary Figure 4).

Next, we correlated the levels of antibody response to the expression of PD-L1 by tumors cells and to the intratumoral T cell infiltrate by performing immunohistochemistry for CD3 and PD-L1. Additional tissue was available for PD-L1 analysis on 39 out of 53 ALK-positive cases. Out of these 39 cases for which tissue was available for further analysis, T-cell infiltrate analysis was possible in 7 out of 9 cases with high ALK antibodies and 22 out of 30 cases with low ALK antibodies. In the remaining 10 cases the material was not adequate for T-cell infiltrate analysis. By these immunohistochemical assays, we could not find a statistically significant difference between patients with high vs low ALK antibodies titers for the amount of T cell infiltrates or the expression of PD-L1 by the tumor cells (Supplementary Figure 5A-5B). The presence of ALK-specific T cell responses in these patients is currently under investigation.

**Figure 3:**
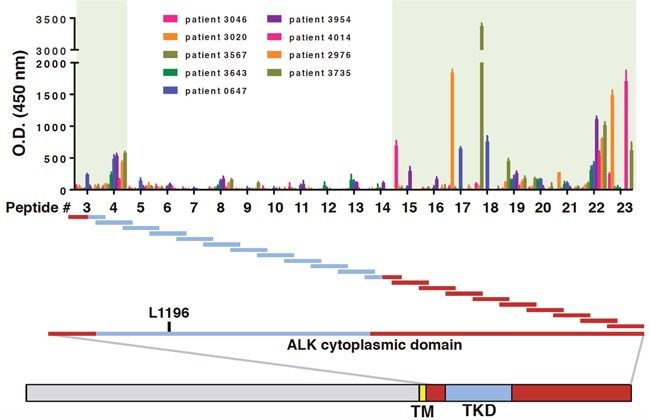
ALK autoantibodies from ALK-positive NSCLC patient recognize different epitopes outside the ALK tyrosine kinase domain Wells in ELISA plates were coated with an array of long peptides, each 36 amino acids in length (peptides #3-23). ELISA O.D. values for nine ALK-positive NSCLC patients with high autoantibody titers are shown. The location of each peptide in relation to the ALK tyrosine kinase domain is shown. For reference, the gatekeeper residue L1196 is also indicated. The shaded green boxes indicate the two regions within the ALK cytoplasmic domain where most of the ALK autoantibodies recognized distinct peptides.

We next sought to more narrowly define the immunogenic peptide sequence(s) of the ALK cytoplasmic domain and determine whether ALK autoantibodies from different patients recognized the same epitopes. To this end, overlapping peptides spanning the entire length of the ALK cytoplasmic domain were synthesized and used to coat wells in an ELISA plate. Each peptide overlapped with the previous one by a 12-amino acid sequence (Supplementary Table 1). While individual serum from the nine ALK-positive NSCLC patients with high ALK autoantibody level detected different portions of the ALK cytoplasmic domain (Supplementary Figure 6), collectively the epitopes clustered mainly N-terminal and C-terminal to the ALK tyrosine kinase domain (Figure 3).

**Figure 4:**
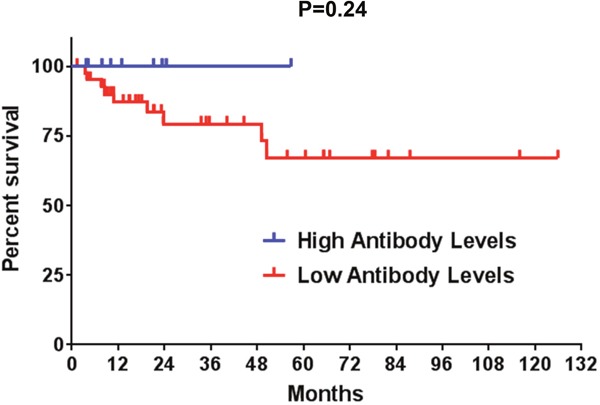
A Kaplan-Meier overall survival analysis is shown for ALK-positive NSCLC patients with high and low anti-ALK antibody levels Censored events are represented as vertical tick marks. High antibody titers (>871.5 O.D.): 9 patients; low antibody titers (≤871.5 O.D.): 44 patients.

Finally, we investigated potential clinical implications of the presence of ALK antibodies in ALK-positive NSLCL patients. While our cohort of ALK-positive NSCLC patients was heterogeneous in terms of their treatment history prior to blood collection, higher ALK autoantibody levels showed a trend toward more favorable overall survival outcomes (P=0.24, Figure 4), although there were several early censoring events in this analysis.

## DISCUSSION

In this work we show that in ALK-positive NSCLC, ALK can be spontaneously immunogenic, albeit in a minority of patients. High levels of ALK antibodies were detected in 17.0% of ALK-positive NSCLC patients by a new quantitative ELISA technique specifically developed for the measurement of ALK antibodies in patient serum. These patients had antibody levels comparable to levels detected in ALK-positive ALCL patients. In contrast, the overall frequency of ALK antibodies appears to be lower than in ALCL patients, in whom anti-ALK antibodies are detectable in up to 96% of patients, with 70% of patients with a titer higher than 1:750, as determined by immunocytochemistry [14]. Further investigation of our whole lymphoma cohort with this newly developed ELISA technique will distinguish whether this higher frequency can be explained by the different methodology used or instead reflects a higher immunogenicity of ALK-positive ALCL compared to NSCLC, possibly because ALK expression in ALCL cells is typically higher than in NSCLC cells [19].

Interestingly, ALK antibodies recognized epitopes which clustered mainly outside the ALK tyrosine kinase domain. This distribution could be explained by the percentage of amino acid identity and similarity of the ALK cytoplasmic portion with other tyrosine kinases. Indeed, amino acid identity and similarity of ALK with the highly related kinases LTK, ROS1 and MET is much higher in the kinase domain than in the C-terminus [20], and this similarity could reduce the probability of developing an immune response against this portion of the protein.

The detection of anti-ALK antibodies in NSCLC raises important questions that should be studied prospectively, such as whether the presence of ALK autoantibodies in ALK-positive NSCLC patients has prognostic significance. It is also unknown whether treatment with chemotherapy, radiation, or TKIs modulates immune recognition of ALK in NSCLC. Our initial analysis on this series of 53 ALK-positive NSCLC patients suggests that the presence of an anti-ALK immune response in NSCLC could be associated with a more favorable prognosis. However, the analysis was limited by the low number of patients and treatment heterogeneity. Larger studies with longer follow-up on a more homogenously treated cohort of patients will be needed to determine whether patients with high ALK autoantibody titers are more likely to be long-term responders to ALK TKIs than patients without detectable ALK antibodies in the serum. It is also possible that these differences could be also related to other factors such as better pre-existing T-cell infiltrates within the tumors of patients with longer survival or additional molecular factors.

Despite an increasing number of clinically-active ALK tyrosine kinase inhibitors, resistance to these agents invariably emerges through several mechanisms. Alternative and novel approaches for treating patients with ALK-positive NSCLC will be necessary to achieve more durable responses. While immune checkpoint inhibitors have revolutionized the treatment of NSCLC, response rates in ALK positive NSCLC patients appear to be low [21]. Whether patients with pre-existing anti-ALK immune responses might be more likely to respond to immune checkpoint inhibitors is currently unknown. There is a growing body of evidence to support the potential development of a cancer vaccine targeting ALK as a shared antigen. In fact, out of a priority-ranked list of 75 tumor antigens evaluated by the National Cancer Institute for cancer vaccine development, ALK ranked fourth [22]. First, as we showed in this study, ALK is a natural shared antigen in NSCLC patients, consistent with previous reports in ALK-positive lymphoma patients [12, 14, 18]. Second, unlike other vaccine strategies that target dispensable tumor antigens, ALK positive lung cancers appear to be dependent on ALK signaling for cell survival, and genomic ALK rearrangements persist even after acquired resistance to ALK inhibitors emerges [6]. Third, expression of ALK is largely restricted to tumor tissue rather than healthy adult tissue [23], which could potentially reduce risk of autoimmunity. Thus, it is possible that stronger immune responses to ALK in NSCLC patients will be associated with improved outcomes, as previously shown for ALK-positive lymphoma patients [14]. To this end, preclinical data show that in mouse models of ALK-rearranged NSCLC, treatment with an ALK vaccine, either alone or in combination with ALK TKIs or PD-1 pathway inhibitors, appears to be a safe and effective approach for prolonging overall survival [16]. Thus, immunotherapeutic strategies that aim at potentiating or generating anti-ALK immune responses could be beneficial in ALK-positive NSCLC patients.

In this context, the present study shows that ALK is indeed spontaneously immunogenic when expressed by non-small cell lung cancer tumor cells. Although we did not find a correlation between ALK antibodies and the amount of intratumoral T-cell infiltrates, the presence of ALK antibodies suggests that an ALK vaccine in human has the potential to elicit immunologic responses. In patients who have already generated an anti-ALK immune response, an ALK vaccine could further potentiate that response, and in patients with no spontaneous anti-ALK immune response, a vaccine might potentially generate a new anti-ALK response that could contribute to an overall anti-tumor immunity. For an effective vaccine therapy, T-cell responses may be more important than antibody responses; therefore, detection of spontaneous T-cell responses in patients with ALK-positive NSCLC will be an important follow-up of this study.

## MATERIALS AND METHODS

### Patients and sample collection

Non-small cell lung cancer patients at the Dana-Farber Cancer Institute were consented to an institutional review board (IRB)-approved correlative research protocol that allowed for review of medical records and sample collection. Lung cancer mutation status was determined using standard CLIA-certified clinical assays in the Center for Advanced Molecular Diagnostics at Brigham and Women's Hospital. For each patient, 10-ml of blood was collected in a serum separator tube, allowed to clot for 20 minutes and centrifuged for 15 minutes at 3000 rpm. Serum was removed and placed into cryovials and stored at -80°C. For positive controls we used serum samples from ALK-positive ALCL patients previously analyzed by an immunocytochemical approach [14].

### Recombinant ALK protein purification and peptide synthesis

Recombinant ALK intracellular domain (ICD, amino acids 1064-1620) was expressed in Sf9 cells as a cleavable GST-tagged product using the BacPAK Baculovirus expression system (Clontech). Cells were infected at a MOI = 5 and harvested after 72 hours, as described [24] and lysed in lysis buffer (50mM Tris-HCl, pH 8.0, 100mM NaCl, 1mM DTT, 0.5mM EDTA, 0.1% Triton-X100, and protease inhibitors: leupeptin, aprotinin, benzamidine, pepstatin A). The total lysate was loaded onto Glutathione Sepharose 4B resin (GE Healthcare) and incubated for 2 hours at 4°C with rotation. After extensive washing, the rALK-ICD was eluted by on-column overnight cleavage by GST-3C protease and concentrated using VivaSpin columns (GE Healthcare, cut-off 10kDa) to a final concentration of 0.3 mg/ml. ALK peptides were synthetized and purified by C S Bio Co., Menlo Park, CA. The full sequence of peptides is presented in Supplementary Table 1. Each peptide was 36 amino acids long, with a 12-amino acid overlap between flanking peptides.

### Measurement of anti-ALK antibodies in serum samples by ELISA

Human anti-ALK antibodies in serum samples were analyzed by a direct enzyme-linked immunosorbent assay (ELISA). 96-well high-bind MAXISORP ELISA plates (NUNC, VWR International, Milan, Italy) were coated with recombinant ALK protein or control bovine serum albumin (BSA, Pierce) at 100 ng/well in 100 μl phosphate-buffered saline (PBS) overnight at 4°C. For ELISA on peptide arrays, each single peptide was coated at 100 ng/well in 100 μl PBS overnight at 4°C. After washing with PBS, non-specific-binding sites were blocked with 200 μl/well of 3% milk-PBS for 3 hours at room temperature (RT). After washing with PBS, 100 μl/well of serum sample were added to each well in triplicates at the dilution 1:200 in 3% milk-PBS and incubated overnight at 4°C. Plates were then washed 3 times with PBS-0.05% Tween 20 and 100 μl/well Horseradish peroxidase–conjugated anti-human immunoglobulin G (Sigma) were used as secondary antibody at 1:2000 dilution in 3% milk-PBS, incubated for one hour at room temperature. After 4 washes with PBS-0.05% Tween 20, plates were developed by incubating 100 μl/well of tetramethylbenzidine substrate (TMB Plus, Nalgene) for 15-30 minutes and the colorimetric reaction was stopped with 50 μl/well 2M hydrochloric acid. Optical density at 450 nm (OD450) was measured using a BIO-RAD microplate reader Model 680 (BIO-RAD, UK). Specific serum sample-ALK binding was calculated by subtraction of non-specific serum sample-BSA mean OD450 value.

### ALK plasmid constructs and 293T transfection

The specific ALK-shRNA cassette to down-regulate NPM-ALK expression was previously described. [25]. Retroviral vectors expressing human NPM-ALK or EML4-ALK were previously described. [26, 27]. The construct for the full length human ALK was cloned in pcDNA3. The ALK extracellular domain (ECD) construct contains the extracellular and transmembrane domains of the human ALK receptor and was amplified by direct PCR from full length ALK and then cloned in pcDNA3 vector at HindIII/XhoI sites.

HEK-293T cells were cultured in Dulbecco's modified Eagle's medium with 10% fetal calf serum (FCS). Transfections of HEK-293T with NPM-ALK, EML4-ALK, full length ALK and ALK ECD constructs were performed with Effectene reagent (Qiagen, Valencia, CA), according to the manufacturer's instructions.

Human lymphoid NPM-ALK positive DHL cells were cultured under standard conditions (37°C in humidified atmosphere, with 5% CO_2_) in RPMI medium (Lonza, Basel, Switzerland) with 10% FCS. Inducible ALK-shRNA DHL (DHL TTA A5) cells were obtained by transduction of pLVtTRKRAB vector followed by pLVTHM vectors containing the cloned ALK-shRNA cassettes. NPM-ALK silencing was achieved after addition of 1 μg/mL of doxycycline to the medium for 72 hours [25].

### Validation of anti-ALK antibodies by western blot

Protein extracts (40 μg) were obtained from cell lysates using GST-FISH buffer (10mM MgCl_2_, 150mM NaCl, 1% NP40, 2% Glycerol, 1mM EDTA, 25mM HEPES pH 7.5, 1mM PMSF, 10mM NaF, 1mM Na_3_VO_4_ and protease inhibitors), separated on SDS-PAGE, transferred to a nitrocellulose membrane, and blotted with patient's serum at the indicated dilutions. Membrane were then incubated with peroxidase–conjugated anti-human IgG (1:1000) and detected by chemiluminescence (ECL, Amersham). To detect the cytoplasmic portion of ALK the rabbit monoclonal anti-ALK antibody D5F3 was used (Cell Signaling Technology). The ALK ECD was detected by specific mouse monoclonal antibody kindly provided by Kolltan [28]. Secondary anti-mouse or anti-rabbit antibodies were purchased from Amersham.

### Immunohistochemistry

Immunohistochemistry was performed on cases with tissue available for further analysis. The following antibodies were used: anti-PD-L1 (clone E1L3N, Cell Signaling Technologies) and anti-CD3 (clone LN10, Leica Biosystems).

### Statistical analysis

Categorical variables were compared using Fisher's exact test. Mean anti-ALK antibody levels were compared using the unpaired Student's t-test method; for patients with serial blood samples drawn over time, the mean O.D. value was calculated and used for each patient. The overall survival analysis was performed using the Kaplan-Meier method using the log-rank test to compare the curves between patients with high or low anti-ALK antibody levels.

## SUPPLEMENTARY MATERIALS FIGURES AND TABLE


