# Supplementary material for: Epitope mapping of spontaneous autoantibodies to anaplastic lymphoma kinase (ALK) in non-small cell lung cancer
Source: Oncotarget. 2017 Sep 23;8(54):92265–74. doi: 10.18632/oncotarget.21182 (PMC5696179; doi:10.18632/oncotarget.21182)
Supplement: Supplementary file 1 [file oncotarget-08-92265-s001.pdf]

Epitope mapping of spontaneous autoantibodies to anaplastic lymphoma kinase (ALK) in non-small cell lung cancer

SUPPLEMENTARY MATERIALS

A

GPLSLDPQELQAMQMELQSPEYKLSKLRTSTIMTDYNPNYCFAGKTSSISDL
KEVPRKNITLIRGLGHGAFGEVYEGQVSGMPNDPSPLQVAVKTLPEVCSEQD
ELDFLMEALIISKFNHQNIVRCIGVSLQSLPRFILLELMAGGDLKSFLRETR
PRPSQPSSLAMLDLLHVARDIACGCQYLEENHFIHRDIAARNCLLTCPGPGR
VAKIGDFGMARDIYRASYYRKGGCAMLPVKWMPPEAFMEGIFTSKTDTWSFG
VLLWEIFSLGYMPYPSKSNQEVLEFVTSGGRMDPPKNCPGPVYRIMTQCWQH
QPEDRPNFAIILERIEYCTQDPDVINTALPIEYGPLVEEEEKVPVRPKDPEG
VPPLLVSQQAKREEERSPAAPPPLPTTSSGKAAKKPTAAEVSVRVPRGPAVE
GGHVNMAFSQSNPPSELHKVHGSRNKPTSLWNPTYGSWFTEKPTKKNNPIAK
KEPHDRGNLGLEGSCTVPPNVATGRLPGASLLLEPSSLTANMKEVPLFRLRH
FPCGNVNYGYQQQGLPLEAATAPGAGHYEDTILKSKNSMNQPGP

GST-vector
ALK sequence (kinase domain)

B

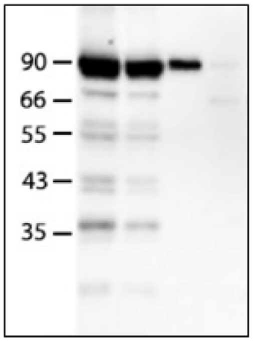

Supplementary Figure 1: (A) The sequence of the recombinant ALK cytoplasmic domain (amino acids 1064-1620) used to directly coat ELISA plates is shown. **(B)** A Western blot using the D5F3 anti-ALK antibody confirms that the purified recombinant GST-tagged ALK protein runs at the expected size of about 85kDa. The lanes represent serial 10-fold dilutions of the recombinant protein (from left to right).

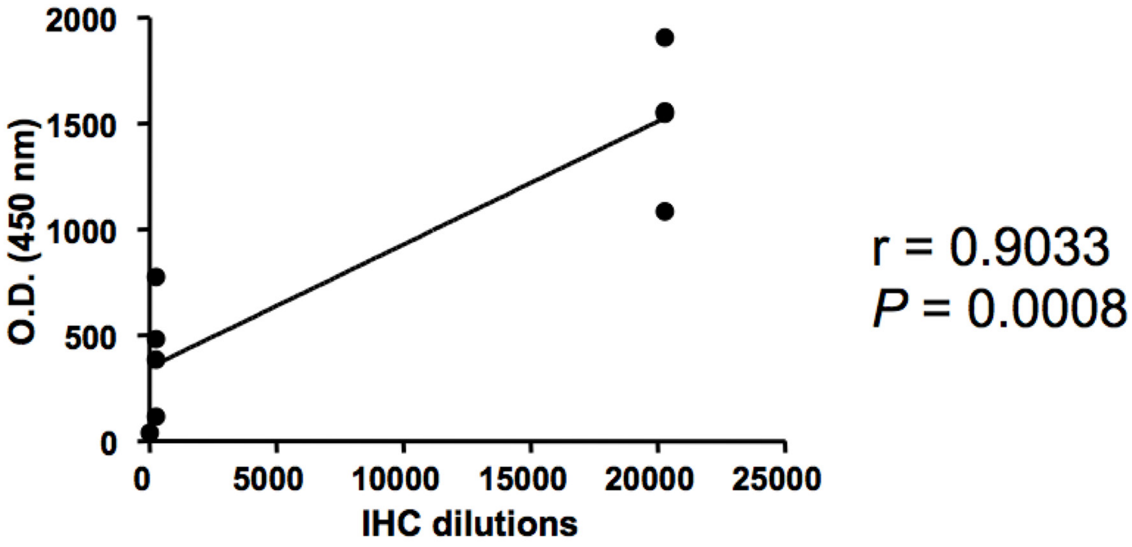

Cases	IHC dilutions	ELISA OD-values
BG	1:20250	1558
DMDS 65	1:20250	1905
DMDS 69	1:20250	1547
XL 85	1:20250	1085
CS	1:250	776
IS	1:250	385
FE 94	1:250	483
XL 96	1:250	117
SL neg	0	40

Supplementary Figure 2: Validation of the ELISA technique to detect anti-ALK antibodies. The OD values obtained by ELISA were correlated with the titer obtained by immunocytochemical technique on the same patients. P value was calculated with two-tailed Pearson correlation.

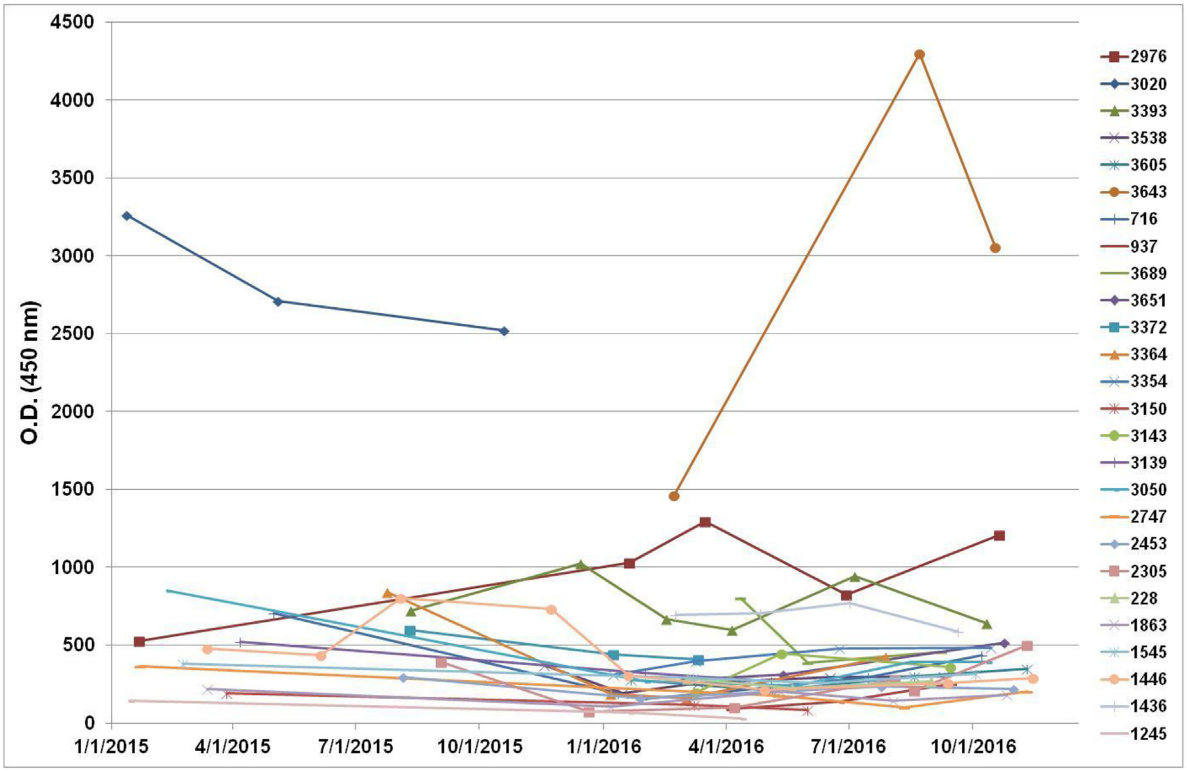

Supplementary Figure 3: High and low ALK autoantibody titers are maintained over time in patients with ALK-positive NSCLC. Of the 53 ALK-positive NSCLC patients, 26 patients (listed by patient ID number on the right) had serial serum samples drawn on at least three different dates. ELISA values for these patients are shown.

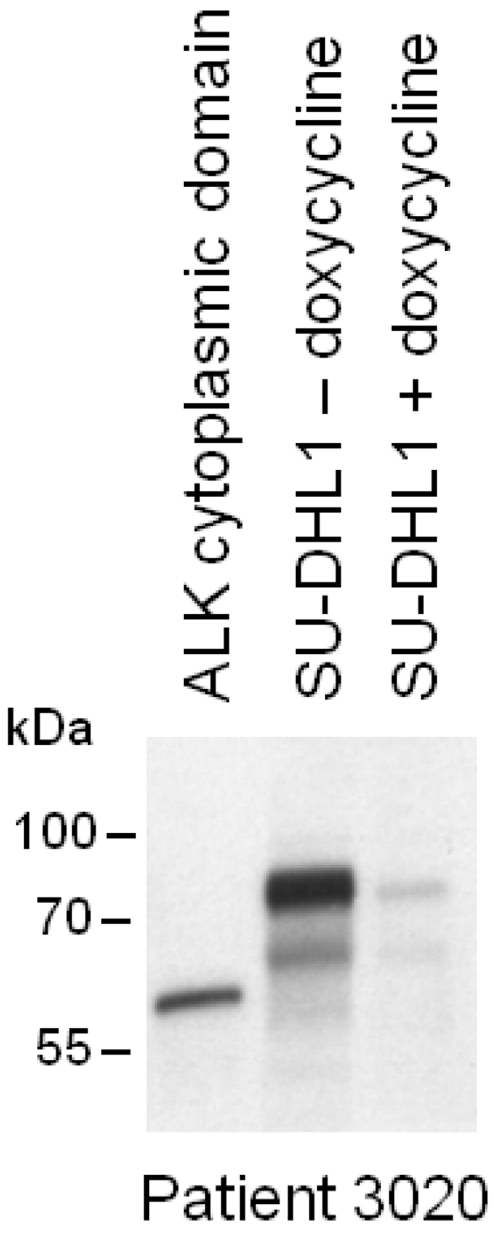

Supplementary Figure 4: Autoantibodies from ALK-positive NSCLC patients are specific for ALK. Serum from patient 3020 was able to detect the recombinant ALK cytoplasmic domain by Western blot (lane 1). The SU-DHL1 NPM-ALK-positive ALCL cell line was transduced with lentivirus containing a doxycycline-inducible ALK shRNA cassette. In the absence of doxycycline (lane 2), NPM-ALK is readily detected, but this signal is markedly diminished in the presence of doxycycline (lane 3).

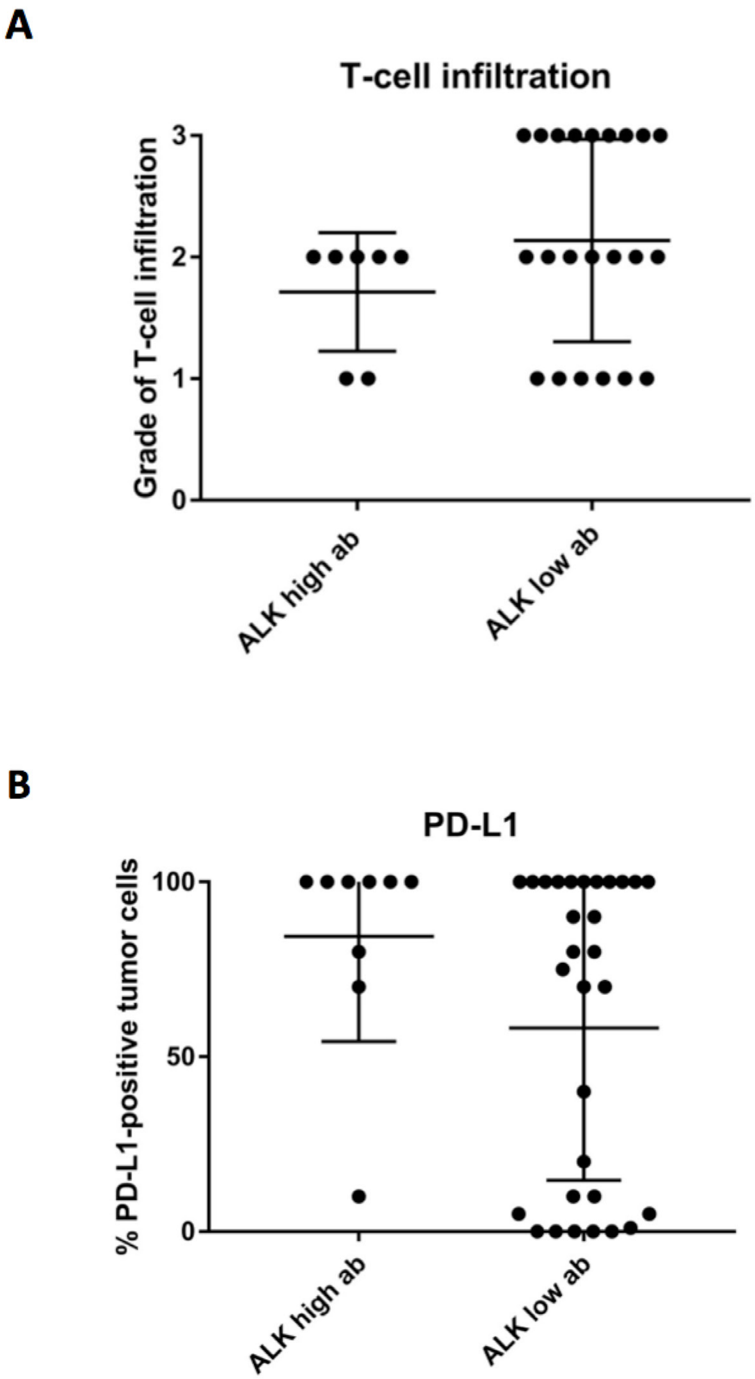

Supplementary Figure 5: Correlation between levels of anti-ALK antibodies and the amount of intratumoral T cell infiltrate **(A)** or the expression of PD-L1 by tumor cells **(B).** Histology sections of patients' tumors were stained for CD3 and PD-L1. The amount of intratumoral T cell infiltrate was evaluated on a three level score based on the percentage of T cells compared to tumoral cells: 1= < 20% T cell infiltrate; 2=20-50% T cell infiltrate; 3= >50% T cell infiltrate. Expression of PD-L1 was measured as percentage of tumor cells positive for PD-L1 staining. T cell infiltrate (A) or PD-L1 expression (B) are shown for ALK+NSCLC patients that have high anti-ALK antibodies vs patients with low anti-ALK antibodies. No significant difference was found between the two groups by Student t test.

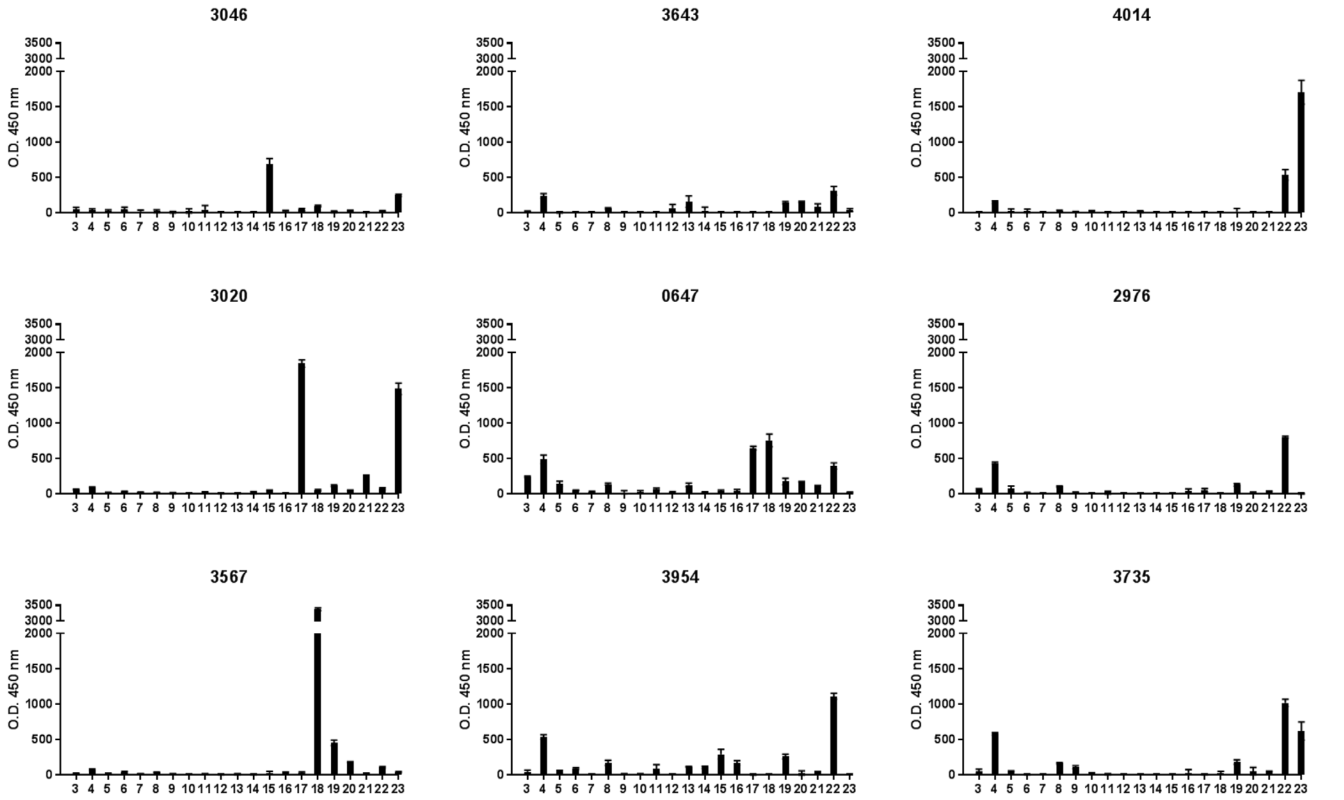

Supplementary Figure 6: Serum from nine different ALK-positive NSCLC patients recognizes distinct peptide epitopes across the ALK cytoplasmic domain, mostly outside the tyrosine kinase domain. Shown are ELISA O.D. values for individual patient serum reacting with each of the tiled ALK peptides (peptides #3-23) which span the entire ALK cytoplasmic omain; data are aggregated in Figure 3. Each patient ID number is listed above each graph.

Supplementary Table 1: Peptide sequences used for ELISA in Figure 3

Peptide Number	Amino Acid Sequence
3	SDLKEVPRKNITLIRGLGHGAFGEVYEGQVSGMPND
4	VYEGQVSGMPNDPSPLQVAVKTLPEVCSEQDELDFL
5	EVCSEQDELDFLMEALIISKFNHQNIVRCIGVSLQS
6	NIVRCIGVSLQSLPRFILLELMAGGDLKSFLRETRP
7	GDLKSFLRETRPRPSQPSSLAMLDLLHVARDIACGC
8	LLHVARDIACGCQYLEENHFIHRDIAARNCLLTCPG
9	IAARNCLLTCPGPGRVAKIGDFGMARDIYRASYYRK
10	ARDIYRASYYRKGGCAMLPVKWMPPEAFMEGIFTSK
11	PEAFMEGIFTSKTDTWSFGVLLWEIFSLGYMPYPSK
12	IFSLGYMPYPSKSNQEVLEFVTSGGRMDPPKNCPGP
13	GRMDPPKNCPGPVYRIMTQCWQHQPEDRPNFAIILE
14	PEDRPNFAIILERIEYCTQDPDVINTALPIEYGPLV
15	NTALPIEYGPLVEEEEKVPVRPKDPEGVPPLLVSQQ
16	PEGVPPLLVSQQAKREEERSPAAPPPLPTTSSGKAA
17	PPLPTTSSGKAAKKPTAAEISVRVPRGPAVEGGHVN
18	PRGPAVEGGHVNMAFSQSNPPSELHKVHGSRNKPTS
19	HKVHGSRNKPTSLWNPTYGSWFTEKPTKKNNPIAKK
20	KPTKKNNPIAKKEPHDRGNLGLEGSCTVPPNVATGR
21	SCTVPPNVATGRLPGASLLLEPSSLTANMKEVPLFR
22	LTANMKEVPLFRLRHFPCGNVNYGYQQQGLPLEAAT
23	GYQQQGLPLEAATAPGAGHYEDTILKSKNSMNQPGP
